# What influences whether researchers adhere to healthcare reporting guidelines successfully? A thematic synthesis

**DOI:** 10.1186/s41073-026-00209-y

**Published:** 2026-07-02

**Authors:** James Harwood, Charlotte Albury, Jennifer de Beyer, Zhaoxiang Bian, Yuting Duan, Shona Kirtley, Michael Schlüssel, Lingyun Zhao, Gary S. Collins

**Affiliations:** 1 The UK EQUATOR Centre, The Nuffield Department of Orthopaedics, Rheumatology and Musculoskeletal Sciences, University of Oxford, Oxford, England; 2 Nuffield Department of Primary Care Health Sciences, University of Oxford, Oxford, England; 3 Chinese EQUATOR Centre, Jockey Club School of Chinese Medicine Building, School of Chinese Medicine, Hong Kong Baptist University, 307 Room, 3/F, HongKong, People’s Republic of China; 4 Department of Applied Health Sciences, School of Health Sciences, College of Medicine and Health, University of Birmingham, Birmingham, UK; 5 NIHR Birmingham Biomedical Research Centre, Birmingham, University Hospitals Birmingham NHS Foundation Trust and University of Birmingham, Birmingham, UK

**Keywords:** EQUATOR Network, STROBE, PRISMA, CARE, CONSORT, COREQ, TRIPOD, SRQR, STARD, SPIRIT, SQUIRE, MDAR, Medical writing, Medical research, Usability

## Abstract

**Background:**

Despite endorsement by medical journals, reporting guidelines have only modestly affected reporting quality in healthcare research. We aimed to identify influences affecting whether authors successfully adhere to reporting guidelines.

**Methods:**

We searched MEDLINE, Embase, PsychINFO, AMED, WHO Global Index Medicus, SciELO, Chinese Biomedical Literature Database, China National Knowledge Infrastructure, Wanfang Data, VIP Chinese Medical Journal Database, OSF, and MiRoR for qualitative research exploring researchers’ experiences of reporting guidelines for healthcare research, published after 1996 in English, Chinese, Spanish, or Portuguese. We appraised studies using CASP-Qual. For thematic synthesis, we applied descriptive codes to all text reporting qualitative findings, then aggregated codes inductively into descriptive themes that captured the codes’ meaning. We interpreted and contextualised possible influences from these descriptive themes to create analytic themes.

**Results:**

From 18 eligible studies, we developed 12 analytic themes: 1) Researchers may not understand guidance as intended or what reporting guidelines are, even if they think they do; 2) Researchers report a variety of reasons for using reporting guidelines, and some are more important than others; 3) Researchers describe using reporting guidelines for different tasks and wanting guidance delivered in ways that better fit their needs; 4) Using reporting guidelines has costs which researchers may feel outweigh benefits; 5) Reporting guidelines may need to be revised and updated; 6) Researchers may not be able to report all items, which can leave them feeling uncertain or worried; 7) Awareness and accessibility may limit reporting guideline usage; 8) Reporting guidelines may be more useful to less experienced researchers, but these researchers may find them harder to use; 9) Researchers want or need design advice, but reporting guidelines may not be the right place to find it; 10) Reporting guidelines can be harder to use if their scope is too broad, too narrow, or poorly defined; 11) Researchers may have to use multiple sets of reporting guidelines, multiplying complexity and costs; 12) Researchers may use checklists but never read the full guidance.

**Discussion:**

We identified many influences despite a paucity of evidence. Addressing these influences when developing, refining, and implementing reporting guidelines may improve adherence.

**Supplementary Information:**

The online version contains supplementary material available at 10.1186/s41073-026-00209-y.

## Introduction

When researchers inadequately describe what they did, why they did it, and what they found, readers find it harder to understand, appraise, replicate, and use research [1]. When information is missing, peer reviewers cannot appraise, researchers cannot replicate, clinicians cannot implement, and patients cannot benefit. When details are omitted, they are lost. The remaining gaps are sources of doubt: do they indicate accidental omissions, oversights in the research itself, or even cover-ups? Whatever their source, the gaps fragment the full picture, and the potential value to patients drains away. For research to be transparent and reproducible, it must be well reported.

Responding to calls for strategies, guides and lists to help authors prepare healthcare research manuscripts, methodologists, trialists, and editors created *reporting guidelines*: recommendations of information to include when writing up research. One of the first and most famous was the CONsolidated Standards of Reporting Trials (CONSORT) statement [2], which comprises a checklist, flow diagram, and (since its 2001 update) ‘Explanation and Elaboration’ publication [3, 4]. CONSORT proved influential, and other groups began publishing reporting guidelines for other kinds of healthcare research. An international group of academics began maintaining a comprehensive online collection of reporting guidelines to help achieve the mission encapsulated in their name: the EQUATOR Network, Enhancing the QUAlity and Transparency Of health Research. EQUATOR Network’s database now includes over 600 reporting guidelines covering different study designs and healthcare topics, as well as studies more peripheral to healthcare like animal research. The reporting guidelines most commonly accessed on the EQUATOR Network’s website include STROBE (Strengthening the Reporting of Observational Studies in Epidemiology [5]), PRISMA (Preferred Reporting Items for Systematic Reviews and Meta-Analyses [6]), and SRQR (Standards for reporting qualitative research [7]).

Journals began endorsing reporting guidelines, and some even made completing a reporting checklist compulsory for manuscript submission. Studies show that reporting has improved since the advent of these guidelines and policies. For example, the reporting of clinical trials has improved since CONSORT was published and journals changed their policies [8, 9]. However, the same studies show that reporting remains far from optimal. Numerous studies show that authors rarely adhere completely to reporting guidelines [10, 11], yet few studies explore *why*. Understanding how and why an intervention is working (or not working) in real life is an important part of designing complex interventions like reporting guidelines, as emphasised by the Medical Research Council’s best practice guidance [12]. However, when auditing the EQUATOR Network database of reporting guidelines [13] we found few reporting guideline development groups had undertaken meaningful user testing (e.g., [14–16]). Most development groups skipped user testing entirely, or barely described it. The few that included qualitative feedback from authors often provided this information as incidental, or anecdotal findings, relegated to discussion sections and supplements.

Scoping searches revealed qualitative reports exploring why authors do not adhere to reporting guidelines were spread thinly across the literature base. Studies mostly focussed on a single reporting guideline or subset, and some explored elements external to reporting guidelines like journal policies of the behaviour of others. There have been no systematic attempts to synthesise these inquiries. Such a synthesis is useful because reporting guidelines are disseminated in similar ways (most commonly as pre-submission checklists), meaning lessons learnt from one reporting guideline are likely to apply to another. Influences must be understood before they can be addressed by reporting guideline developers, the EQUATOR Network, or other stakeholders. Understanding and addressing influences are the first steps towards improving adherence to reporting guidelines, and thereby improving the quality of medical research reporting.

Our aim was therefore to identify influences affecting authors’ adherence to reporting guidelines for healthcare research by synthesising qualitative reports of authors’ experiences of using these guidelines. Whereas health behaviour researchers often write about “Barriers and Enablers” or “Barriers and Facilitators”, we use the word *influence*. We found our data did not fall neatly into these binary categories: What hindered one person sometimes helped another, or what helped in one scenario did not help in another. Haynes and Loblay [17] address this struggle and argue against the use of “barriers and facilitators,” which they describe as an “untheorised framing device that often rests on unexamined assumptions yet, because it is familiar, is chosen as a ‘safe’ approach,” perhaps because it makes “qualitative research appear more palatable to reviewers.” Haynes and Loblay argue that a barrier and enabler approach is a false friend; “If it can claim any theoretical basis,” they argue, “it would be that the approach is loosely underpinned by a linear-rationalist behavioural paradigm,” but this simplified linearity can lead to superficial findings that do not consider context nor encourage reflexivity. Following leading behavioural scientists (e.g., [18]), we instead use “influence,” defined by the Oxford dictionary as something that has “capacity to have an effect on the character, development, or behaviour of someone or something.”

## Methods

To identify influences affecting whether authors adhere to reporting guidelines, we performed a thematic synthesis of qualitative research that explored researchers’ experiences of reporting guidelines.

### Researcher roles and characteristics

As thematic synthesis involves subjective interpretation, we reflected on our opinions, beliefs, and experiences so that we, and our readers, could be aware of how our stances may colour our collection, interpretation, and description of data.

All authors except CA are affiliated with the EQUATOR Network, which promotes reporting guidelines. All authors consider incomplete research reporting to be a common and important problem and believe reporting guidelines and checklists to be useful tools. GSC is Director of the UK EQUATOR Centre and an author of the TRIPOD guidelines for clinical prediction models, GATHER statement for global health estimates, AGReMA for mediation analyses, TIDieR-placebo for placebo-controlled trials, RIPL checklist for patent landscapes, and member of the MISTIC, STARD-AI, TRIPOD + AI, DECIDE-AI, CONSORT-AI, SPIRIT-AI, CONSORT-Surrogate, and REMARK, CONSORT and SPIRIT update working groups. SK is an author of the PRISMA-S guidelines for reporting systematic review literature searches. JdB assisted on the 2024 CONSORT and SPIRIT updates. ZB is Director of the Chinese EQUATOR Centre. ZB and YD have helped develop reporting guidelines for Chinese medicine and have translated existing guidelines into Chinese, including nine CONSORT extensions, the ARRIVE guidelines, and the SPIRIT extension for patient-reported outcomes. MMS is leading studies that assess reporting completeness in health research based on the contents of several reporting guidelines checklists, the robustness of reporting guidelines development methods, and the consolidation of different reporting guidelines for the reporting of studies of nutritional interventions. JH is the creator of an automated manuscript checker [19] that recommends reporting guidelines to authors. His experience in software development led him to question the usability of reporting guidelines.

### Approach to searching and data sources

Our search strategy sought all research publications reporting qualitative data exploring researchers’ experiences of using reporting guidelines. We included international databases in our information sources to capture the experiences of researchers from around the world. Alongside searching databases and websites, JH looked through EQUATOR’s database audit [13], emailed reporting guideline developers, and performed forwards and backwards citation searches. All data sources are listed in Table 1.

**Table 1:** Information sources and record management

Source	Search platform	Date searched
Medline	Ovid	08/12/2021
Embase	Ovid	08/12/2021
Allied Complementary Medicine Database (AMED)	Ovid	08/12/2021
PsycInfo	Ovid	08/12/2021
Latin American and Caribbean Health Sciences Literature [20]	WHO Global Index Medicus (GIM) [21]	08/12/2021
African Index Medicus [22]	WHO Global Index Medicus (GIM)	08/12/2021
Western Pacific Region Index Medicus [23]	WHO Global Index Medicus (GIM)	08/12/2021
Index Medicus for South-East Asia region [24]	WHO Global Index Medicus (GIM)	08/12/2021
Index Medicus for the Eastern Mediterranean Region [25]	WHO Global Index Medicus (GIM)	08/12/2021
Scientific Electronic Library Online [26]	https://scielo.org/en/	08/12/2021
Open Science Framework (OSF)	https://osf.io/	15/12/2021
Methods in Research on Research website [27]	http://miror-ejd.eu/publications/	14/12/2021
Emailing developers of guidelines listed in Table 1	n/a	08/01/2022
Forward and backward citation searching	n/a	08/01/2022
Chinese Biomedical Literature Database [28]	Chinese Biomedical Literature Service System [29]	08/12/2021
China National Knowledge Infrastructure [30]	https://www.cnki.net/	08/12/2021
Wanfang Data [31]	http://www.wanfangdata.com/	08/12/2021
VIP Chinese Medical Journal Database [32]	http://www.cqvip.com/	08/12/2021

### Inclusion and exclusion criteria

We included published research articles reporting researchers’ experiences of using reporting guidelines derived through qualitative methods. We included studies where the reporting guideline covered healthcare research, or preclinical research including animal research and laboratory studies. Although the EQUATOR Network works hard to maintain its comprehensive catalogue, reporting guidelines can evade its notice if reporting guideline developers do not register their resource. Therefore, reporting guidelines did not have to be included in the EQUATOR Network database to be included in this study. We excluded articles published before 1996, the year the CONSORT statement was first published, as CONSORT is the oldest reporting guideline still in use. We were able to screen studies written in Chinese, Spanish, Portuguese, and English. We excluded articles written in any other language during screening.

We included articles reporting feedback from researchers as part of reporting guideline development. We decided not to include reporting guideline development studies where this feedback came exclusively from development group members, as we considered this context to be too different to how ordinary researchers experience reporting guidelines. In mixed-method surveys, we did not consider categorical survey questions with a free text option for “other” to be qualitative, but we did include findings from free text questions inviting participants to provide context to a previous (not qualitative) question.

### Electronic search strategy

The UK EQUATOR Centre’s information specialist (SK) helped develop our comprehensive search strategies, which had components for reporting guidelines and qualitative methods. We constructed the reporting guidelines component by combining acronyms of frequently accessed guidelines and generic terms for reporting guidelines. These generic terms ensured our search captured reporting guidelines that were not explicitly named within the search, whether or not they appear in the EQUATOR Network database. Our search did not seek quality appraisal tools.

Our qualitative component came from a review of search filters [33], which recommended a sensitive qualitative filter for systematic reviews [34]. We extended the filter to include descriptive methods to capture target records using mixed-method surveys. We conducted scoping searches, but our search strategies were not peer reviewed before execution and we did not set up article alerts. Our search strategies are reported fully in Additional file 1.

### Screening

One author (JH) used Zotero to deduplicate records. He then used Rayyan [35] to screen titles and abstracts to identify articles exploring researchers’ experiences of reporting guidelines, before screening full texts to identify whether those articles used qualitative methods. Another author (MS) double-screened a random 10% sample using their titles and abstracts. Differences occurred for only three studies, and agreement was reached through discussion after comparing the full text against the eligibility criteria. A member of the Chinese EQUATOR Centre (YD) used Zotero to deduplicate and screen Chinese records in the same way, asking her colleague (LZ) for second opinions when necessary. As the eligibility criteria were clear and objective we did not pilot our inclusion criteria, nor attempt to calibrate decisions between reviewers.

### Describing and appraising records

One author (JH) extracted study characteristics and used the Critical Appraisal Skills Programme Qualitative (CASP-Qual) checklist [36] to critically appraise included studies. We expected this appraisal to help us consider strengths and weaknesses of each study when synthesising them.

### Synthesis methodology

We used thematic synthesis as defined by Thomas and Harden [37] because it can handle studies with “thin” descriptions, it allowed us to infer influences from research that may not have addressed our research question directly, and we expected its output, grouped by themes, to be useful to healthcare reporting guideline developers.

One author (JH) imported files into NVivo 12.0 for Mac [38] and coded all text from the results section and relevant supplementary materials that reported qualitative findings. He assigned each sentence one or more descriptive codes that sought to distil the essence of what was written, creating new codes when necessary and without using a framework. He then used mind-mapping software [39] to visualise similarities and differences between codes and aggregate them inductively into descriptive themes. These themes were descriptive in that they captured the meaning of the codes they contained. He then used the research question to infer influences from these descriptive themes, thereby producing analytic themes. We used peer checking to enhance the credibility of our analysis; JH met with a co-author (JdB) three times through the coding process. JH and JdB discussed all coded text, descriptive codes, descriptive themes, and analytic themes and resolved coding decisions through discussion. We did not prioritise themes by frequency because we expected code frequency to be biased by the questions asked in each study.

We noted when a code pertained to a particular reporting guideline and report this connection when it gives context to a quotation or finding. However, we chose not to categorise codes by reporting guidelines as our objective was to explore researchers’ experiences of reporting guidelines in general, not to compare themes between reporting guidelines, as reporting guidelines are disseminated in similar ways. Additionally, we expected some studies would ask about reporting guidelines in general, or would not report which reporting guideline a participant was referring to.

### Reporting guidelines

When writing this article, we used the PRISMA-S guidelines for reporting systematic searches (Additional file 1) and the ENTREQ guidelines for reporting qualitative evidence syntheses (Additional file 1) [40, 41].

### Changes to protocol

As we expected to find quantitative and mixed-method surveys alongside purely qualitative studies, we initially planned [42] to consider both quantitative and qualitative data, inspired by Thomas et al. [43]. However, combining primary and secondary order constructs was theoretically complex, and emulating Thomas et al*.*’s 203-page report within the constraints of a journal article was not feasible. Instead, we reviewed the content of non-qualitative survey questions in a separate study [44]. Articles that collected both qualitative and non-qualitative data (n = 14) were included in both studies, but the data were synthesised separately: qualitative data is synthesised in this study, non-qualitative in the other. For example, where mixed methods studies asked both Likert-style and open-ended questions, we analysed only the open-ended responses here. The same author (JH) coded the non-qualitative questions for the other study after analysing the qualitative data reported here so as to avoid being sensitized by them, but before writing this article so that comparisons between the non-qualitative questions and qualitative data could be discussed.

## Results

### Search

**Fig. 1:**
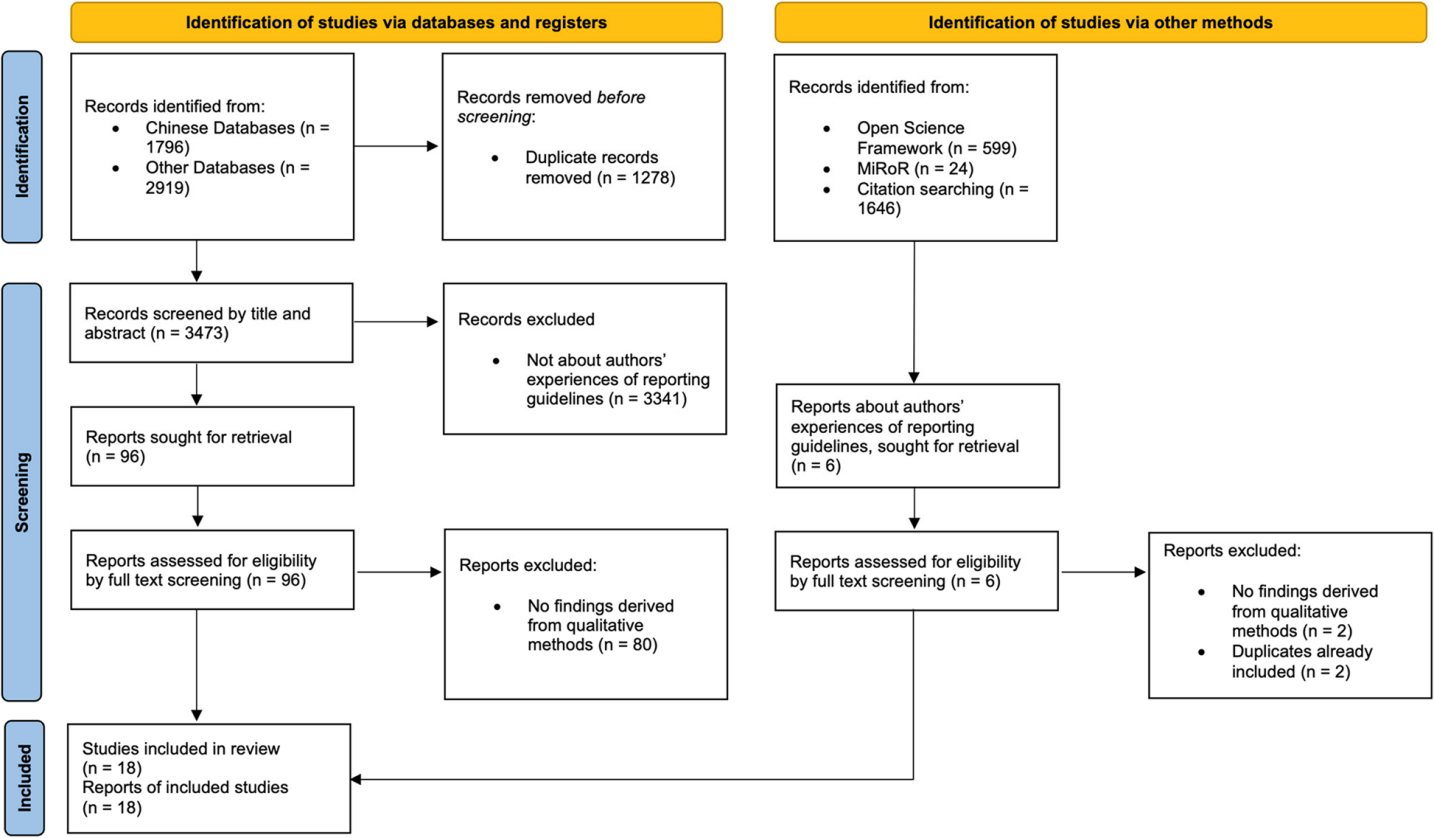
PRISMA flow diagram

Our search yielded 18 articles (see Fig. 1 for full search results). JH and MS double-screened 10% of the non-Chinese titles and abstracts and agreed on 98.3% of them (170/173). They resolved the remaining three through discussion and consensus. Citation searches, emails with reporting guideline developers, and the EQUATOR database audit did not reveal additional articles. All eligible records were in English; there were no eligible articles written in Chinese, Spanish, or Portuguese. Eligible articles included surveys, semi-structured interviews, focus groups, and writing tasks, and are summarised in Table 3.

The 18 included articles investigated 12 reporting guidelines (see Table 2). Ten of the 18 looked at one or more well-known guidelines whose acronyms were included in our search strategy (e.g., ARRIVE, CONSORT, PRISMA, SQUIRE, STARD, and STROBE). Our search also yielded studies examining lesser-known reporting guidelines that were not named in our search like TREND, the Standards for Reporting Interventions in Controlled Trials of Acupuncture, and the Materials Design Analysis Reporting framework. The latter was the only reporting guideline not already included in the EQUATOR Network’s database. These reporting guidelines covered a range of common biomedical research designs (e.g., clinical trials, observational epidemiology), primary research and systematic reviews, research on humans, animals and in life sciences (see Tables 2, 3). Two articles explicitly investigated reporting guidelines in general (without naming any in particular) and another did not specify which reporting guidelines respondants were referring to.

**Table 2:** Reporting guidelines evaluated using qualitative methods in the studies synthesised in this review

Acronym	Full Name	Types of research covered
CONSORT	Consolidated Standards of Reporting Trials	Reporting of randomised trials
STROBE	Strengthening the Reporting of Observational Studies in Epidemiology	Observational studies in epidemiology (cohort, case–control studies, cross-sectional studies)
PRISMA	Preferred Reporting Items for Systematic Reviews and Meta-Analyses	Reporting systematic reviews and meta-analyses
STARD	STAndards for Reporting Diagnostic accuracy studies	Studies of diagnostic accuracy
SQUIRE	Standards for QUality Improvement Reporting Excellence	Quality improvement in health care
TREND	Transparent Reporting of Evaluations with Nonrandomized Designs (TREND) statement	Reporting of intervention evaluation studies using nonrandomized designs
MDAR	Materials Design Analysis Reporting	Framework for transparent reporting in the life sciences
STRICTA	Revised STandards for Reporting Interventions in Clinical Trials of Acupuncture	Interventions in clinical trials of acupuncture
ARRIVE	Animal Research: Reporting of In Vivo Experiments	Reporting any area of bioscience research using laboratory animals
PRISMA Equity	PRISMA-Equity 2012 Extension: Reporting Guidelines for Systematic Reviews with a Focus on Health Equity	Systematic reviews with a focus on health equity
CONSORT Ehealth	Consolidated Standards of Reporting Trials of Electronic and Mobile HEalth Applications and onLine TeleHealth	Evaluation reports of Web-based and mobile health interventions
n/a	Systematic review protocols of animal intervention studies	Protocols for systematic reviews of animal intervention studies

**Table 3:** Study characteristics

Authors	Participants	Participants’ country	Guidelines	Methods	Phenomena of interest	CASP rating
Burford et al., 2013 [45]	151 systematic review authors	Not reported	PRISMA Equity	Mixed-methods survey	Perceived utility, facilitators, and barriers	Fairly valuable
Davies et al., 2015 [46]	18 experts and 29 end users	USA, Canada, Sweden, the UK, and Norway	SQUIRE	Focus groups and interviews	Experiences with and impressions of the SQUIRE Guidelines	Valuable
Davies et al., 2016 [14]	44 graduates faculty, and directors of healthcare	Not reported	SQUIRE	Mixed-methods survey and written exercise	“whether SQUIRE 1.6 was understood and implemented as intended by the developers”	Valuable
de Vries et al. 2015 [47]	7 researchers	Not reported	Systematic review protocols of animal intervention studies	Participants were asked to give feedback, but it is unclear whether this feedback was given in an interview or in writing	Feedback on usability, missing items, possibilities for improvement and clarity	Not very valuable
Dewey et al., 2019 [48]	74 out of 831 survey respondents provided (optional) free-text comments	The full survey was answered by respondents in the USA, Canada, China, South Korea, Japan, Germany, France, Italy, UK, other European countries, Middle East, Latin America, other. It is unclear where respondents for the free-text answers came from	CONSORT, STROBE, PRISMA, STARD	Mixed-methods survey	“(1) When and how are reporting guidelines and checklists used by authors and reviewers? (2) What is their impact on the content of final manuscript drafts according to authors? and (3) How do authors and reviewers perceive the value of reporting guidelines and related checklists?”	Fairly valuable
Eysenbach 2013 [49]	61 authors	Not reported	CONSORT Ehealth	Mixed-methods survey	Views on completing the checklist as part of submission	Fairly valuable
Fuller et al., 2015 [50]	5 authors	USA and Australia	TREND and Reporting Guidelines in general	Survey and semi structured interviews	Factors that affected authors’ use of TREND and other reporting guidelines	Valuable
Korevaar et al., 2016 [51]	4 radiology residents, 8 laboratory medicine experts	Radiology residents were from the Netherlands. No geographical details provided for experts	STARD	Interview (residents) and mixed-methods survey (experts)	To identify items that were vague, ambiguous, difficult to interpret, or missing	Fairly valuable
Macleod et al., 2021 [52]	211 authors, but only some answered the free-text question	The full survey was answered by participants in the USA, China, Japan, Germany, EU, and “Other” areas. It is unclear who answered the free-text question	Materials Design Analysis Reporting framework	Mixed-methods survey	Whether the checklist was clear and useful	Fairly valuable
Page et al., 2021 [53]	110 systematic review authors and experts	Not reported	PRISMA	Mixed-methods survey	Opinions on PRISMA and proposed changes	Valuable
Prady et al., 2007 [54]	40 authors	Not reported	Standards for Reporting Interventions in Controlled Trials of Acupuncture	Mixed-methods survey	Experiences using PRISMA	Fairly valuable
Prager et al., 2021 [55]	5 of 18 survey respondents that answered the free-text question	Not reported	STARD	Mixed-methods survey	Barriers to STARD 2015 adherence	Fairly valuable
Rader et al., 2014 [56]	263 systematic reviewers	Not reported	PRISMA	Mixed-methods survey	Barriers or difficulties in meeting more detailed reporting standards in PRISMA	Fairly valuable
du Sert et al., 2020 [57]	11 authors	UK, USA, Belgium, Brazil	ARRIVE	Interview and writing task	Authors’ opinions, interpretation, and experiences of updated ARRIVE guidelines	Fairly valuable
Sharp et al., 2020 [58]	203 of 1015 researchers that answered free-text questions	The full survey was answered by participants in Africa, Asia, Europe, North and South America, Middle East, and Pacific Region. It is unclear who answered the free-text question	STROBE	Mixed-methods survey	Experiences and attitudes towards STROBE	Valuable
Struthers et al., 2021 [59]	623 authors, 274 of whom answered free the text question	Not reported	Reporting guidelines in general	Mixed-methods survey	The question asked, “What could I do to improve the guideline?”	Fairly valuable
Svensøy et al., 2021 [60]	10 authors	Not reported	Not specified	Semi structured interviews	Experiences using guidelines or templates	Valuable
Tam et al., 2019 [61]	230 authors, 62 of whom answered the open-ended questions	Not reported	PRISMA	Mixed-methods survey	Opinions on PRISMA	Fairly valuable

Only 7 of the 18 records reported where participants came from. Three mixed-method survey studies [48, 52, 58] included participants from a wide range of countries but it was not possible to tell which participants completed the optional qualitative questions. Three interview studies [46, 50, 51] included participants exclusively from North America, Europe, and Australia, whilst the fourth [57] included a participant from Brazil.

Using CASP-Qual for critical appraisal, we rated the studies from valuable to not very valuable. We did not exclude the less valuable studies as they had few qualitative components or minimal reporting of qualitative analysis or findings, and so naturally contributed fewer codes. Study characteristics are reported in Table 3.

### Synthesis findings

The relationships between our codes, descriptive themes, and analytic themes are reported in Table 4. We identified the following analytic themes: 1) Researchers may not understand guidance as intended or what reporting guidelines are, even if they think they do; 2) Researchers report a variety of reasons for using reporting guidelines, and that some are more important than others; 3) Researchers describe using reporting guidelines for different tasks and wanting guidance delivered in ways that better fit their needs; 4) Using reporting guidelines has costs which researchers may feel outweigh benefits; 5) Reporting guidelines may need to be revised and updated; 6) Researchers may not be able to report all items, which can leave them feeling uncertain or worried; 7) Awareness and accessibility may limit reporting guideline usage; 8) Reporting guidelines may be more useful to less experienced researchers, but these researchers may find them harder to use; 9) Researchers want or need design advice, but reporting guidelines may not be the right place to find it; 10) Reporting guidelines can be harder to use if their scope is too broad, too narrow, or poorly defined; 11) Researchers may have to use multiple sets of reporting guidelines, multiplying complexity and costs; 12) Researchers may use checklists but never read the full guidance.

**Table 4:** Codes (left, not bold), descriptive themes (left, bold), and analytic themes (right)

DESCRIPTIVE THEMES CODES	ANALYTIC THEMES
**Unclear meaning** A term’s meaning can be unclear [14, 46, 51, 53, 59] An item’s meaning can be unclear [14, 46, 51, 53, 54, 59] The difference between items can be unclear [46, 49, 53, 54] Researchers can misunderstand text [14, 46] Examples can help researchers understand [47, 53, 57] **Unclear importance** An item’s importance may be unclear [46, 51, 53, 61] It may not be clear *who* an item is important to [46, 53, 58] **Unclear applicability** Understanding the guideline’s scope as intended [53, 59] Understanding whether an item applies [46, 49, 53, 54, 59] Understanding whether an item is optional [46, 54] **Understand reporting guidelines at an abstract level** Understanding what reporting guidelines are [55, 58] Understanding how to use a reporting guideline? [50]	Researchers may not understand the guidance as intended, or what reporting guidelines are, even if they think they do
**Personal benefits of reporting guidelines** Finding reporting guidelines useful in general [48, 59] Reporting guidelines make users feel confident [58] Reporting guidelines help users develop as researchers [45, 58] Reporting guidelines help users improve manuscripts [45, 46, 48, 49, 58] Believing reporting guidelines may ease publishing [60] **Using reporting guidelines because of other people** Using reporting guidelines because of instruction from journals and editors [45, 50, 58, 60] Using reporting guidelines because other researchers expect it [50, 60] **Reporting guidelines benefit others** Standardised reporting benefits the community [58, 60, 62] **Some benefits are more important than others** Immediate benefits are more important than hypothetical ones [58, 60] Personal benefits are more important than benefits to others [60]	Researchers report a variety of reasons for using reporting guidelines, and that some are more important than others
**Researchers use reporting guidelines for different tasks** Using reporting guidelines for planning research [46, 58] Using reporting guidelines for designing research [48, 54, 55, 58] Using reporting guidelines for writing [46, 48, 54, 58] Using reporting guidelines for checking their own or other people’s writing [55, 58] Using reporting guidelines to appraise the quality of other people’s reporting [51] Using reporting guidelines for peer reviewing [58] **Wanting reporting guidance presented in formats that are better suited to the task at hand** Wanting items presented in the order they must be done [47, 62] Wanting design or methods advice [46, 53, 58] Wanting templates for writing [48] Wanting checklists that are easy to fill in [52, 59] Wanting checklists embedded into journal submission workflows [48] Wanting items embedded into data collection tools [45]	Researchers report using reporting guidelines for different tasks and wanting guidance to be delivered in ways that better fit their needs
**Reporting guidelines take time** Reporting guidelines take time to read, understand and apply [45, 50, 60] Some items require extra work which takes time and effort [14, 46, 56] Wanting an indication of which items to prioritise [46, 54] Perceived complexity [46, 48, 52, 60] Long guidelines are off-putting [45, 49, 58, 59] **Itemization may decrease costs** Itemization helps navigating guidance [53] Itemization summarises the guidance [48] **Itemization may increase perceived costs** Itemization makes guidance appear longer [53] Itemization blocks the bigger picture [46] **Reporting guidelines make manuscripts long and bloated** Following reporting guidance can result in long, bloated articles [45, 46, 49, 54] Long, bloated articles may exceed journal word limits [49, 50, 52, 54] Wanting options for where to report items [14, 46, 49, 50, 53, 58] **The benefits of using a reporting guideline may not outweigh the costs** The benefits of using a reporting guideline may not outweigh the costs [49, 50, 58] **The balance of benefits vs costs may be more favourable when reporting guidelines are used early** Reporting guidelines are more valuable when used early [46, 48, 58, 59]	Using reporting guidelines has costs, and researchers may not feel that benefits outweigh the costs
**Opinions on how guidance could be improved** Clarify an item [53, 54] Move an item [14, 46] Split an item into two [46, 47, 53] Adding or remove items [46, 51, 53, 54] Adding or removing requirements from an item [53, 54, 57, 58, 61] **Reporting guidelines need to be kept updated** Reporting guidelines can become out of date [46] Reporting guidelines need to be updated [53]	Reporting guidelines may need to be revised and updated for different reasons
**Being unable to report something** Unable to report something because it wasn’t done [46, 49, 53, 54] Unable to report something because of intellectual property issues [49] Unable to report something because it clashes with journal guidelines [53] Unable to report something because data was missing from primary studies [45] Editors, reviewers or co-authors requested removal of an item [54, 56] **Feeling nervous or uncertain when unable to report an item** Feeling uncertain because of not knowing how to say that they didn’t do something [53] Feeling worried about being judged for transparently reporting something they didn’t do [53, 58]	Researchers may not be able to report all items which can leave them feeling uncertain or worried
**Researchers can only use what they know about and have** Researchers may not know that reporting guidelines exist, or what guidance exists [48, 50, 51, 59, 60] Researchers may not be able to easily access guidance [59, 60]	Awareness and accessibility may limit reporting guideline usage
**Reporting guidelines are more valuable to inexperienced researchers** Reporting guidelines may be less valuable to experienced researchers [48, 49, 58] Experienced researchers feel that they already know how to report [46, 48, 58] Experienced researchers find guidance patronizing and feel untrusted [49, 50, 52, 53] **Reporting guidelines can be hard to use at first but get easier with experience** Reporting guidelines can be hard to use at first but get easier with experience [46, 50, 60]	Reporting guidelines may be more useful to less experienced researchers, but less experienced researchers may find them harder to use
**Wanting or needing design advice** Wanting design or methodological advice [52, 53, 58] Now knowing how to do an item [46, 53, 54] **Thinking reporting guidelines prescribe how research should be designed** Reporting guidelines are procedural straightjackets [58] Reporting guidelines are too prescriptive [53, 58, 61]	Researchers want or need design advice, but reporting guidelines may not be the right place
**A reporting guideline’s scope can be unclear** Reporting guideline’s applicability criteria are not clear [48, 51, 59] **A reporting guideline can be too narrow** There isn’t always a perfect reporting guideline for every study [59] Some reporting guidelines don’t generalise [48, 52, 53, 58, 61] **A reporting guideline’s scope can be too broad** Researchers don’t want to see optional items that only apply to other types of study [54, 59]	Reporting guidelines can be harder to use if their scope is too broad, too narrow, or poorly defined
**Researchers often need to adhere to multiple sets of guidance** Researchers need to adhere to journal guidelines or other research guidelines [48, 50, 53, 54] Researchers might need to use multiple reporting guidelines [58] **Researchers want guidelines to harmonise** Researchers want reporting guidelines to be linked or embedded [51, 53] Researchers want reporting guidelines to use similar structure [53] Researchers want reporting guidelines to use similar terms [53]	Researchers may have to use multiple sets of reporting guidelines, multiplying complexity and costs
**Researchers experience reporting guidelines primarily as, or through, checklists** Researchers don’t like checklists [48, 49, 58, 59] Researchers may use the checklist instead of the full guidance [57]	Researchers may use checklists but never read the full guidance

As mentioned in the introduction of this chapter, barriers and facilitators were not spoken about as consistent experiences. What may be a barrier for one person might be an enabler to another or when occurring in a different context. We therefore do not label our analytic themes as one or the other and instead describe them as *influences*.

#### Researchers may not understand guidance as intended or what reporting guidelines are, even if they think they do

Researchers commonly stated that they need more information to fully understand the intention of the reporting guideline developer. When asked about the clarity of guidance, researchers across many studies reported difficulty in understanding certain terms, concepts, or checklist items:

*“Does [the word outcome] mean the domain, or does it mean the domain measure, metric, method of aggregation, and time? *[53]*.”*

*“Primary and secondary improvement related question is confusing, what does that mean?… I had a hard time with the [difference between the] improvement question and the study question.” *[46]

A few researchers reported ignoring an item if they could not understand it:

*“Only one item was identified as hard to understand by more than one respondent: ‘methods employed to ensure completeness of data’, which two participants said they left out because of difficulty in comprehending the item” *[14]

Some researchers reported feeling that reporting guidelines were “simply not comprehensible.” [46] Others reported that they had understood, but further investigation revealed that their interpretations could be “different from that intended by the developers.” [14] For example, Davies et al. [14] found that one SQUIRE item “was reported as used fully [in the quantitative survey], but the qualitative analysis revealed that its usage was frequently inconsistent with the intention of the developers.” Researchers may interpret the guidance in different ways depending on their prior experience, the research context, or if the guidance is ambiguous. For example, the SQUIRE developers found that the word ‘theory’ “meant different things to different people. For some, the word ‘theory’ meant ‘mechanism by which an intervention was expected to work’, for others it meant ‘lean or six sigma for example’, and for still others it meant ‘logic model’.” [46]

Even when researchers reported understanding what an item meant, they may not have understood why it is important or who it is important to, leading them to remark that an item “seems unnecessary.” [57] Few researchers referenced the needs of evidence synthesisers or patients as consumers of research, but more reported considering whether an item would be useful to other researchers, editors, and reviewers:

*“the information provided does not matter as the reviewers do not know what to do with it’’*[58]

Many researchers also expressed difficulty understanding whether an item was applicable to their work. Some reporting guidelines specify that not all items are compulsory or that some items may only apply to a subset of research articles. Researchers highlighted that this nuance may not be obvious, especially if buried in a long elaboration document. Some researchers therefore reported uncertainty over which items applied to them:

*“Authors asked for clarification of which items were always required and which were nonessential” *[54]

*“Not always clear what was relevant to their study” *[57]

*“He had realised with experience and re-reading the Guidelines that SQUIRE did not require him to include every item in the manuscript.”*[46]

This uncertainty may extend to the entire reporting guideline if researchers don’t know when to use one over another. One researcher declared that “PRISMA guidelines can also be used rather than the MOOSE,” [59] when the two are primarily for reviews of intervention studies and observational studies respectively. Sometimes there may not be a perfect reporting guideline for a given study, as one researcher commented after using ARRIVE (which focusses on experimental research involving laboratory animals):

*“Our report was an animal based cadaveric study looking at accuracy of drill guides. We were unsure which category it should fall under.” *[57]

Even if a researcher understands the guidance, why it is important, and why it applies to them, they may not understand how to report information or “how much detail to report.” [57] Some researchers “used examples [included in the guidance] to understand what should be reported” because they “demonstrate what is meant in practice.” [57]

At a more fundamental level, researchers varied in their understanding of what reporting guidelines are. Often researchers would talk about reporting guidelines as if they were design guidelines, e.g., describing STROBE as “woefully deficient in encouraging…use of appropriate data analytic approaches.” [58] This criticism suggested that the researcher had not noticed the stipulation that “these recommendations are not prescriptions for designing or conducting studies” included in STROBE’s explanation and elaboration document [63]. Other researchers wrote about STARD as is if the guidance was to be used when collecting imaging data:

*“Two comments suggested that reporting quality may be impacted by the physical environment in which […] data are collected. These comments may indicate an incomplete understanding of reporting guidelines which pertain to reporting results during manuscript writing, not the process of imaging acquisition itself.” *[55]

#### Researchers report a variety of reasons for using reporting guidelines, and that some are more important than others

Some researchers listed personal benefits to using reporting guidelines. Some described reporting guidelines as a “training tool” [45] for personal development, noting that guidance helps “develop a strong foundation and habits.” [58] Some talked about how guidance made them feel: “As a junior scientist it gives me confidence to request the reporting of a certain piece of information.” [58] Others said that reporting guidelines are “a helpful reminder” [45] and that going through the checklist “improved their manuscripts” [49]. Some saw value in fostering a “transparent reporting process” [48] and for making sure your “project is written up […] rigorously.” [46]

Some researchers noted altruistic benefits: that widespread reporting guideline adherence “helps in standardizing how research is reported” [58] and calling “for more scientific reports to be published, preferably using a template or guideline to make them comparable.” [60]

In the absence of anticipated benefits, some researchers said they use reporting guidelines simply because “it was what was implicitly expected of them to do.” [50] These expectations came from journals and their peers. Some used “tools promoted by journals, which often promised to ease the publishing process” [60] but others wrote that they found this to be an empty promise:

*‘’I have never had (nor have I heard of) an editor or reviewer pushing back on a claim that all STROBE criteria were met. Therefore, when a STROBE checklist is required for manuscript submission, it seems to turn into a[n] exercise in additional administrative busywork without really improving the research.’’*[58]

A few researchers reported being more likely to comply with journal requirements if they thought the journal was likely to enforce them: “Does the journal only suggest or actually require submission of a reporting guideline checklist?” [50] Some said they were more likely to comply if “it was a high impact factor journal and I thought that I would only get one crack at it.” [50]

A few researchers compared different motivations for using reporting guidance, noting that personal, guaranteed, and immediate benefits were more motivating than hypothetical benefits or benefits to others:

*“I suppose you are looking for short-term gain, short-term benefits as a writer of a report” *[60]

*“it can be difficult to put the energy into using STROBE (or any other) one a priori since ultimately, it depends on the journal submitted to and accepted to” *[58]

*“All the researchers wanted more homogenous reporting but emphasised that: ‘As an individual reporter, one is prone to choose the easiest and most accessible one.’” *[60]

#### Researchers describe using reporting guidelines for different tasks and wanting guidance delivered in ways that better fit their needs

Although reporting guidelines were designed to help researchers draft and check their manuscripts, many researchers mentioned using reporting guidelines for other tasks, such as designing research, planning, peer-reviewing, and educating. A few researchers suggested ways that the guidance could be delivered to make their tasks easier. For example, some “thought [the] order of items should reflect [the] order considered when designing the experiment.” [57] Others wanted “a manuscript template” [48] to make writing easier. Some suggested that “online form[s]” [52] or software to “mark in the text what corresponds to each item in the list” [59] would make it easier to complete a reporting checklist as part of journal submission.

#### Using reporting guidelines has costs which researchers may feel outweigh benefits

Researchers noted that some items require extra work, either to collect the necessary information or just to think about and report, and that sometimes this workload felt overly burdensome:

*“If [reporting guideline developers] put the onus on everybody out there who’s trying to improve care to deal with that sophisticated question […], I just think [they] are putting a barrier in place that is going to be a mountain” *[14]

This work requires time. The “length of time it would take to consider the items” [45] was cited by many researchers as a cost, with some asking themselves whether “sufficient time [was] available to comply with [the] reporting guideline.” [50]

Researchers noted that a reporting guideline’s “length and content is a key factor influencing the time needed to complete it.” [58] Some found checklists to be “very complete, but to follow every single point is overwhelming” [49]. As a solution, many wanted to “simplify” or “shorten the checklists.” [59] A few researchers wanted a “hierarchy” to know which items were most “important to include.” [14] Another suggested that checklists presented as online forms could include “logic for irrelevant [items]” [52] so that the users are presented with only items that apply to them.

Complexity was sometimes mentioned alongside time: “As the research often was performed out of work hours, the required time and complexity of the guidelines or templates may have played a crucial role [in deciding whether to use a reporting guideline].” [60] Researchers had conflicting opinions about whether itemization reduces perceived complexity. Proponents noted that the “Checklist is a very helpful summary of sometimes confusing guidelines” [48] and that itemization made guidance “easier to follow” and “more approachable.” [53] But a few said that presenting guidance in small pieces made it difficult to “get the whole picture of what you are supposed to be doing” [46] and that itemization makes “the checklist appear more daunting for users” because it adds vertical length. Consequently, “If you make the checklist too long people will see it as too complicated and then won’t use it.” [53]

Another concern cited by many researchers was that following a reporting guideline can result in long reports:

*“I use SQUIRE a lot for planning—I complete the sections up through the methods at the time I design the study…[but] SQUIRE creates sort of long reports if followed exactly.” *[46]

*“the document you create if you use SQUIRE exactly as written is unintelligible” *[46]

*“this [item] would require another paper” *[49]

This problem was exacerbated by journal word limits:

*“I believe it is a useful instrument but it is unrealistic to assume that every single suggestion can be detailed in a 6000-words manuscript.” *[49]

*“two remarked that word limitations has necessitated removal of many items” *[54]

Although a handful of researchers noted that “the relaxation of word limits” [50] would help, many researchers objected to long articles because they were bloated, harder to read, or simply “unintelligible” [46] and requested strategies to “enhance readability” regardless of journal policies. Some wondered where they could place this information outside of the article body, such as “in an appendix,” an “online supplement or repository,” or a figure [53]. Some researchers preferred to report information in the checklist instead of the article body because of “space restrictions, because [it was] a minor component of the study, because they considered the information to be obvious, or because they were unsure of how to incorporate it in the manuscript.” [57] Some used this strategy to report items that had “not been used or observed during the study, for example that no inclusion or exclusion criteria had been set, no data had been excluded, randomisation and blinding had not been used…” [57] although it was not clear whether this was motivated by a desire for a concise article or a concern about highlighting potential weaknesses.

Faced with the costs of time, work, and article length, some researchers explicitly weighed perceived benefits against costs and disagreed about the balance:

*“The manuscript has improved. However, I felt that the amount of effort was considerably greater than the degree of improvement.” *[49]

*“it also adds to the time required to put together a manuscript, and I am not sure how much it improves the chances of a manuscript being published” *[58]

*“it does increase the quality of the articles, it is clearly worth the time” *[58]

The balance of costs versus benefits may be most favourable when guidance is used early in the research workflow. Researchers who used reporting guidelines earlier in their workflow (e.g., for planning research or drafting) used language that implied it was something they did regularly (e.g., “I use SQUIRE a lot for planning” [46]). Some reported that they had come to this habit by their own initiative and that reporting guideline developers should “encourage people to use the criteria early in the writing process (I have, which probably is why I only changed one thing [at the point of submission]).” [59] One researcher suggested that “policy that focuses on a front end approach would be helpful,” [58] noting that “To fully apply the criteria, I would need to systematically apply the STROBE criteria on the front end design of a project, grant, etc. rather than at the time of writing a project.” [58]

Conversely, many authors who completed a checklist during manuscript submission, very late in their in workflow, emphasised the costs, using words like “arduous” [58] and expressing negative opinions of this process (see *Researchers may use the checklist but never read the full guidance*). This may be because researchers lack the motivation, time, or ability to edit their manuscripts at this point.

#### Reporting guidelines may need to be revised and updated for different reasons

Researchers in most studies had opinions on how guidance could be improved through clarifying, reorganising, splitting, merging, adding, or deleting items, and sometimes these views fed into the revision of reporting guidelines [14, 53]. This feedback may be useful for reporting guideline developers. Even if a reporting guideline was considered perfect at one point in time, researchers noted that guidance must be kept up to date in response to changes in the field and broader scientific ecosystem:

*“The evolution of the healthcare improvement scholarly literature in the intervening years since the publication of the SQUIRE Guidelines has led to the development of concepts that were not fully anticipated at the time of initial release.” *[46]

Updates to one reporting guideline may necessitate the update of another. For instance, as PRISMA was being updated, a few researchers “supported referring to PRISMA for Abstracts, but suggested it also needs updating” to reflect updates being made to PRISMA [53].

#### Researchers may not be able to report all items, which can leave them feeling uncertain or worried

Some researchers described being unable to report items because of external factors, including intellectual property or data rules, disagreement between co-authors, or because “peer reviewers or editors had suggested editing out much of their [reporting guideline]-specific text.” [54] Others reported feeling unable to report an item because they did not do it, whether on purpose, due to an oversight, or because requirements had changed since the study began:

*“[This item was] not part of the study objectives” *[49]

*“This [item] is a good idea, but I did not do this.” *[49]

*“The RCT was initiated before trial registration became customary in Norway, and therefore does not have a Trial ID number.” *[49]

This left some researchers fearing that “an incomplete checklist [gave] the impression that their study is less than perfect.”[58]. Some expressed concern that strict wording that assumed something was done may “force people to lie/mislead by asking a question they cannot answer” [53] and suggested that guidance should instead use more agnostic language and specify what to do if an item were not addressed, such as “If no publicly accessible protocol is available, please state this.” [53]

#### Awareness and accessibility may limit reporting guideline usage

Researchers may not know what guidance exists and may be more likely to use whatever is most accessible and discoverable:

*“Several of the researchers did not have extensive knowledge about the different reporting tools, so the accessibility of the guideline or template was often a decisive factor.” *[60]

One researcher wrote that “poor dissemination strategy by authors of reporting guidelines had inhibited uptake,” [50] and others recognised that reporting guidelines could be “better highlighted” [48] by journals or advertised on “social media platforms.” [59]

#### Reporting guidelines may be more useful to less experienced researchers, but these researchers may find them harder to use

Some researchers reported that they didn’t need the guidance as they were experienced enough to know what they were doing:

*“One of the most prevalent themes was the expression of self-assuredness. ‘[I] follow the STROBE guidelines in my reporting reasonably well without actually referring to them or using a checklist’ (group 3, ID1) and ‘[I] already apply the STROBE recommendations despite not having heard of it until today’” *[58]

Sometimes this was accompanied by an acknowledgement that reporting guidance may be more beneficial to less experienced researchers:

*“Despite experienced researchers generally not seeing a benefit to personally using STROBE, there were strong feelings that it is valuable to early-career researchers” *[58]

*“Helpful at beginning of career, but not at later stage” *[48]

*“this exercise might be good for college students but is insulting for professionals” *[49]

However, less experienced researchers often reported finding “reporting guidelines being difficult to use initially,” [50] or that a reporting guideline became easier after repeated use, after using other reporting guidelines, or with experience in medical writing in general. For instance, “Participants with less experience in scholarly medical writing found the SQUIRE Guidelines harder.” [46]

#### Researchers want or need design advice, but reporting guidelines may not be the right place

Many researchers reported wanting advice on design choices but disagreed on where that design guidance should go. Some researchers suggested referring researchers to other design resources through hyperlinks or citations. Others explicitly wanted design guidance to be written into reporting guidelines so that others would read it. Some went as far as calling for reporting guidelines to express an opinion and encourage one technique over another. One researcher objected to a “neutral tone” [53] in a reporting guideline that may give the impression that a design choice (that they disapproved of) was reasonable practice. Although this could be interpreted as a participant confusing the role of reporting guidelines with quality appraisal tools, it could also be because some reporting guidelines (including well known ones like ARRIVE) purposefully embed design advice, in the hope that researchers will consult the reporting guideline whilst planning a study or writing a protocol.

However, other researchers objected to reporting guidelines that were opinionated about design choices. One user described STROBE as a “procedural straightjacket,” [58] suggesting that it dictates how studies should be conducted. Users who encounter the guidance late in writing may be unable to act on any design recommendations and consequently may feel fearful of reporting transparently if their design choices deviate from what the guideline recommends as best practice (see *Researchers may not be able to report all items, which can leave them feeling uncertain or worried*).

Perhaps with these concerns in mind, one wrote about the “need to make sure that the language around this elaboration gives [researchers] some flexibility,” [53] with another noting they were “OK with the idea of emphasizing the value of [this design choice], but [they would not] mandate it.” [53]

These comments suggest some participants may perceive reporting guidelines as study design/methodological guidance. This perception may be due, in part, because some well-known reporting guideline development groups (e.g., ARRIVE) purposefully embed design guidance in their reporting guideline in the hope that researchers will consult it whilst planning a study or writing a protocol.

#### Reporting guidelines can be harder to use if their scope is too broad, too narrow, or poorly defined

Reporting guideline developers may narrow the scope of their guidance by limiting it to certain design choices or research contexts. This frustrated some researchers, who noted that narrow “checklists cannot fit all types of research” [48] and “cautioned that ‘balance between freedom and structure is important to consider’ […] and that it is ‘important to recognise that each study/analysis is unique and doesn’t always fit with the recommendations’.”[58]

Scope may not always be clearly communicated. One PRISMA user opined that “the assessment of risk of bias, statement of risk ratio and explaining additional analyses depend on the study design … [For] a systematic review of cross-sectional surveys or a meta-synthesis I do not need this information,” [61] suggesting they were unaware of PRISMA’s focus on interventional studies or that MOOSE and ENTREQ would be more appropriate for these kinds of studies (see previous themes for further discussion of awareness and understanding the applicability of reporting guidelines).

Researchers noted that scope could be made broader by removing items or, more commonly, by extending items with more options and examples:

*“omit”(benefits or harms)” from the checklist item to be more inclusive of reviews that do not examine effects of interventions” *[53]

*“If the new PRISMA will more explicitly embrace topics other than interventions (which I think it should), then some additional examples could be added to the parenthesis (e.g., sensitivity and specificity, disease prevalence, regression coefficient)” *[53]

However, extending guidance with options can make the guidance appear longer and means researchers must work out which parts apply to them.

#### Researchers may have to use multiple sets of reporting guidelines, multiplying complexity and costs

There are now over 600 reporting guidelines indexed on the EQUATOR Network website, with more added each year as reporting guideline developers seek to cover more use cases. Researchers may be expected to use a second or third reporting guideline alongside the original one. Reporting guidelines that are intended to be used in addition to another are called extensions. Some researchers “pointed out that these extensions have created needless complexity and additional confusion in reporting of observational studies […] and that the number of extensions has become excessive, especially given that multiple extensions may apply to a single study.” [58]

One researcher wrote: “it would be good to have better connection between different checklists (perhaps using digital linking, decision-trees, etc.).” [53] Some showed concern that hyperlinks to extensions will go unused and so developers should “incorporate all relevant details in the […] checklist and elaboration (in case authors don’t read the extension).” [53] When writing about PRISMA, one researcher noted that “it would be wise to limit the number of additional documents to look up. This is only item 7, and I have already been referred to PRISMA for Abstracts and PRISMA for Searches. As a systematic review author, reviewer, or editor, I would be unlikely to go to several sources for reporting guidance.” [53]

A few researchers wrote that related reporting guidelines should be mutually updated to keep in sync with each other before linking or embedding them. Researchers wanted the instruction, terminology, and structure of different sets of reporting guidelines to be coherent, suggesting, for example, that the updated PRISMA should be structured to be “in line with PRISMA-P [PRISMA for protocols].” [53]

Researchers must also comply with journal, funder, and other scientific guidelines and expressed frustration when instructions contradicted each other. For example, some reporting guidelines specify subheadings for abstracts and one researcher pointed out that a “major issue is that journals wildly differ in requirements/what is allowed in abstracts.” [53]

#### Researchers may use checklists but never read the full guidance

Reporting guidelines typically consist of the guidance itself and a checklist that serves as a summary of the guidance and a tool to demonstrate compliance. Sometimes the document containing the full guidance is called the Explanation and Elaboration (or E&E for short). When talking about a reporting guideline, it was often unclear whether the researcher was talking about the checklist or the E&E.

Some researchers implied that their only experience with reporting guidelines was completing a checklist as part of submission. Many negative statements were directed specifically at this process, describing checklists as “painful”, [48] “pedantic”, “annoying”, [58] or a “stupid exercise.” [49]

One study explored researchers’ use of checklists and E&E documents, noting that “Participants used the guidelines and the E&E in different ways. Some did not read the E&E and used only the checklist, others read the E&E first and then used the checklist and a further group used the checklist and referred to the E&E for help with specific items.” [57] One researcher even went as far as to say that the “E&E appeared to be redundant.” [57]

If some researchers only use checklists, which typically lack any nuance included in the E&E, this may explain why some described reporting guidance as inflexible and prescriptive, warning that “Blind checklists are not relevant to most work” [59] or that “Authors may fear the ‘Checklist Manifesto’ becoming a rigid bureaucracy, and also becoming contrived.” [58]

## Discussion

The aim of this study was to identify influences that may affect whether researchers adhere to reporting guidelines when writing up their work. Our synthesis revealed that researchers describe many challenges when trying to use reporting guidelines and have many questions, opinions, and suggestions that could be useful for reporting guideline developers. Researchers also report personal benefits, especially when using a reporting guideline early in their research projects. These benefits are at odds with how reporting guidelines are typically disseminated (as pre-submission checklists) and presented (as a benefit to others). The polarity and severity of these influences differ according to context (e.g., when or how a reporting guideline is used) and personal characteristics (e.g., experience in academic writing).

These findings could help increase the impact of reporting guidelines if taken into consideration during their development, dissemination, and implementation. Guideline developers, frustrated when authors do not adhere to their guidance, have previously called for journals to better enforce the use [64] of reporting guidelines. However, these results suggest that focussing on enforcement alone is short-sighted. Most identified influences could be addressed by reporting guideline developers directly, without involving journals or editors. Doing so may in turn make it easier for journals and funders to enforce reporting guidelines. For example, it is difficult to enforce a reporting guideline that is complicated to understand or if the guideline’s applicability criteria are unclear.

Our aim was to identify influences, but not to identify solutions. Sometimes solutions may seem obvious. However, we would urge reporting guideline developers to think through solutions carefully and thoroughly. When we used the results of this study to guide our own redesign of the EQUATOR Network website and reporting guidelines [65], behaviour change frameworks (e.g. [66]; [67]) helped us to consider options systematically.

Many of the influences we identified came from respondants talking about well-established reporting guidelines that were developed in line with the EQUATOR Network’s guidance for reporting guideline developers [68], which is considered best practice for developing reporting guidelines. Hence the barriers to uptake identified in this study are not confined to poorly developed guidelines, and may persist even when reporting guideline developers follow EQUATOR’s recommended, evidence-informed development processes.

This suggests that the EQUATOR Network guidance for reporting guideline developers [68] needs to encourage reporting guideline developers to conduct user-testing, as many pitfalls could be avoided through feedback, for example to determine whether readers interpret guidance as intended. First published in 2010, EQUATOR’s guidance covers steps from inception to dissemination but largely neglects user experience or user testing. Only two short sections, totalling eight sentences of an eight-page document, address the importance of gathering user feedback. The guidance offers no instruction on how to do this such as what methods to consider, ways to recruit, and how to justify and cost user testing in grant applications. This may help explain why a recent audit of reporting guidelines in the EQUATOR Network database found that few undergo piloting at all and that pilots are rarely reported in detail [13]. It may also explin why we only found 12 reporitng guidelines had undergone qualitative user testing, and why these qualitative components were often small, perhaps limited to a single question like “Please add your comments and suggestions in the free text below” [48] or “Any other feedback?” [52].

## Limitations

Our listed themes should not be taken as exhaustive. We have already described how our review is limited by the availability of literature and the relative thinness of some studies’ qualitative analysis. Most studies may have been subject to recall bias as they relied on participants recalling what they had done or thought in the past. Future studies could consider using ‘in the moment’ methods like think-aloud tasks.

Qualitative data from mixed-method surveys will be shaped by proceeding questions. In our review of non-qualitative questions [44], we found overlap between influences explored by those questions and themes identified in the qualitative data. However, we feel confident that the qualitative findings presented here are not mere artifacts of question order bias. Firstly, influences 6 and 8–12 were not explored by any non-qualitative questions. Secondly, where influences did appear in both qualitative data and non-qualitative questions, the qualitative data offers a much richer exploration of that influence. For example, although some surveys explored personal benefits (e.g., [69]), some benefits only appeared in the qualitative data (e.g., confidence [58] and personal development [45, 58]). Thirdly, the qualitative data often went beyond non-qualitative questions by offering explanations to them. For example, although survey questions explored whether reporting guidelines were hard to understand, only the qualitative data revealed what, exactly, was hard to understand (or misunderstood): a word, an item, scope, applicability, etc.

Although we tried to capture the experience of a diverse range of researchers by searching international databases, most participants were from Western countries. Our Chinese database searches yielded no relevant studies, and we found no studies on this topic published in Spanish or Portuguese. Around a quarter of visitors to the EQUATOR Network’s website have their browser set to a language other than English, and non native-English speaking researchers may well face additional challenges not covered here. The studies synthesised in this chapter were all conducted in English. This may explain why language barriers did not appear as a theme, despite being explored as a potential issue in quantitative surveys (e.g., [55]).

We did not distinguish between different reporting guidelines. Our objectives were not to compare reporting guidelines, nor to explore one reporting guideline in particular, but to find themes that may generalise. However, readers should not assume that all themes apply to all reporting guidelines equally and we have refrained from stipulating which themes pertained to any given reporting guideline. We hope reporting guideline developers will consider whether the themes we identified apply to their reporting guidelines, and perhaps use our findings as a starting point for their own enquiry when developing or refining a reporting guideline.

If we were to attempt a comparative analysis in the future, instead of comparing themes between reporting guidelines, we would be more interested to explore differences between contexts (e.g., researchers’ experiences of using reporting guidelines early vs. late in their writing process) or between qualitative and quantitative researchers. It was notable that reporting guidelines for qualitative research have never been evaluated qualitatively. Given the variation in epistemologies and methodologies amongst qualitative researchers, it is probable that researchers using these guidelines may face unique challenges.

Our searches were conducted in 2021, a few years before publication. As this study was part of JH’s DPhil thesis and received no dedicated funding, the searches could not be updated before submission. Nevertheless, the themes we identified are still valid. Any qualitative evidence published in the interim would serve to extend the themes we identified, provide extra context, or may contribute additional themes, but would not make the themes we identified redundant.

## Reflections

This study was the first of JH’s PhD. Before CA joined as a co-author, the rest of the author team had no experience in qualitative synthesis and we were not sure which reporting guideline to use (ironic, given where we work). We settled on using the Journal Article Reporting Standards for Qualitative meta-analyses (JARS-Qual)[70]. Developed by the American Psychological Association, it felt like a good fit as our phenomena (experiences of using a reporting guideline) fell within the realm of social sciences. However, when we tried to publish our protocol, the editor (who came from the medical meta-research world) insisted we use ENTREQ, [41] which was developed for reporting syntheses of *health* research, even though our phenomena was not health-related. Although we felt ENTREQ was less relevant to our work, there was sufficient overlap to switch. Neither ENTREQ nor JARS-Qual have ready-to-use checklists, so we had to make our own, and we found parts of ENTREQ confusing. Aware that we were experiencing some of the influences described in this study, we took extra care to avoid giving undue emphasis to challenges we experienced ourselves.

## Conclusions

Researchers encounter many influences that may affect whether they adhere to reporting guidelines. Guideline developers could address many of these influences when developing, refining, and implementing their resources. Few reporting guidelines have been evaluated qualitatively, and many of these evaluations suffer from thin description, recall bias, question order bias, and lack diverse sampling. Reporting guideline developers should be encouraged, supported, and funded to evaluate their resources using in-depth qualitative methods.

## Supplementary Information

Additional file 1.

## Data Availability

Screening and coding data have been deposited on the OSF https://osf.io/wt5f9/

## Abbreviations

CASP: Critical Appraisal Skills Programme
ENTREQ: Enhancing Transparency in Reporting the Synthesis of Qualitative Research statement
EQUATOR: Enhancing the QUAlity and Transparency Of health Research
GIM: Global Index Medicus
OSF: Open Science Framework
PRISMA-P: Preferred Reporting Items for Systematic Reviews and Meta-Analyses Protocols
PRISMA-S: Preferred Reporting Items for Systematic Reviews and Meta-Analyses Searches
PRISMA Equity: Preferred Reporting Items for Systematic Reviews and Meta-Analyses with a Focus on Health Equity
SciELO: Scientific Electronic
