# Supplementary material for: What influences whether researchers adhere to healthcare reporting guidelines successfully? A thematic synthesis
Source: Res Integr Peer Rev. 2026 Jul 2;11:33. doi: 10.1186/s41073-026-00209-y (PMC13326295; doi:10.1186/s41073-026-00209-y)
Supplement: Supplementary file 1 — Additional file 1. [file 41073_2026_209_MOESM1_ESM.docx]

Online Supplement

James Harwood, Charlotte Albury, Jennifer de Beyer, Zhaoxiang Bian, Yuting Duan, Shona Kirtley, Michael Schlüssel, Lingyun Zhao, Gary Collins. *What influences whether researchers adhere to reporting guidelines successfully? A thematic synthesis.*

### Table of Contents

[Supplement 1: Search strategies 1](#_Toc209435886)

[Supplement 2: PRISMA-S Checklist 4](#_Toc209435887)

[Supplement 3: ENTREQ Checklist 7](#_Toc209435888)

## Supplement 1: Search strategies

We did not seek any external peer review of our search. We did not record the database versions at the time of searching due to an oversight. We performed forward and backwards citation searching but found no additional records. We did not set up email alerts.

### Ovid search strategy

Databases: Medline, Embase, AMED, PsycINFO.

Search date: 08/12/2021

We used a federated search. The *kw* field does not exist in PsycINFO or AMED and so was ignored by these databases. The *tw* field does not exist in AMED either and was mapped to *af* instead.

1. ((reporting or writ$ or author$) adj2 (checklist$ or statement$ or guid$ or template$ or standard$ or recommendation$)).ti,kw.
2. ((consort$ or strobe$ or stard$ or prisma$ or moose$ or squire$ or arrive$ or remark$ or tripod$ or cheers$ or spirit$ or srqr$ or coreq$) adj3 (guid$ or statement$ or checklist$)).ti,kw.
3. (experience$ or interview$ or survey$ or questionnaire$ or "focus group$" or facilitat$ or barrier$).af.
4. qualitative.tw.
5. 1 or 2
6. 3 or 4
7. 5 and 6

Platform-specific filter applied: 1996 – current year

### Global Index Medicus & SciELO search strategy

Databases: Latin American and Caribbean Health Sciences Literature, African Index Medicus, Western Pacific Region Index Medicus, Index Medicus for South-East Asia Region, and Index Medicus for the Eastern Mediterranean Region, searched using Global Index Medicus (https://www.globalindexmedicus.net/); Scientific Electronic Library Online (<https://scielo.org/en/>).

Search date: 08/12/2021

ti:(((reporting OR writ* OR author*) AND (checklist* OR statement* OR guid* OR template* OR standard* OR recommendation* OR experience* OR interview* OR survey* OR questionnaire* OR "focus group*" OR facilitat* OR barrier* OR qualitative*)))

Platform-specific filter applied: 1996 – current year

### Chinese Biomedical Literature Database

Database URL: <https://www.imicams.ac.cn/>

Search Date: 25/10/2021

1. 报告 OR 撰写 OR 作者

2. 清单 OR 声明 OR 指导 OR 规范 OR 指南 OR 共识 OR 模板 OR 标准 OR 推荐意见

3. CONSORT OR PRISMA OR STROBE OR SPIRIT OR STARD OR SRQR OR ARRIVE OR SQUIRE OR CHEERS OR TRIPOD OR COREQ

4. 经历 OR 体验 OR 访谈OR 调查 OR 问卷调查 OR 焦点小组 OR 焦点群众

5. 促进 OR 阻碍

6. 质性研究 OR 定性研究

7. (#2) AND (#1)

8. (#7) OR (#3)

9. (#6) OR (#5) OR (#4)

10. (#9) AND (#8)

11. ((#9) AND (#8)) AND ("循证文献"[文献类型] OR "临床试验"[文献类型])

### China National Knowledge Infrastructure

Database URL: <https://www.cnki.net/>

Search Date: 25/10/2021

( ( ( TI = '报告' OR TI = '撰写' OR TI = '作者') AND (TI = '清单' OR TI = '声明' OR TI = '指导' OR TI = '规范' OR TI = '指南' OR TI = '共识' OR TI = '模板' OR TI = '标准' OR TI = '推荐意见' ) ) OR ( TI = 'CONSORT' OR TI = 'STROBE' OR TI = 'PRISMA' OR TI = 'SPIRIT' OR TI = 'STARD' OR TI = 'SRQR' OR TI = 'ARRIVE' OR TI = 'SQUIRE' OR TI = 'CHEERS' OR TI = 'TRIPOD' OR TI = 'COREQ' ) ) AND (TI = '经历' OR TI = '体验' OR TI = '访谈' OR TI = '调查' OR TI = '问卷调查' OR TI = '焦点群众' OR TI = '焦点小组' OR TI = '促进' OR TI = '阻碍' OR TI = '质性研究' OR TI = '定性研究' )

### Wanfang Data

Database URL: http://www.wanfangdata.com/

(((题名或关键词:(报告 or 撰写 or 作者)) and (题名或关键词:(清单 or 声明 or 指导 or 规范 or 指南 or 共识 or 模板 or 标准 or 推荐意见))) or (题名或关键词:(CONSORT or STROBE or STARD or PRISMA or MOOSE or SQUIRE or ARRIVE or REMARK or TRIPOD or CHEERS or SPIRIT or SRQR or COREQ))) and (题名或关键词:(经历 or 体验 or 访谈 or 访问 or 采访 or 调查 or 问卷调查 or 焦点小组 or 焦点群众 or 促进 or 阻碍 or 质性研究 or 定性研究))

### VIP Chinese Medical Journal Database

Database URL: http://www.cqvip.com/

(((M=(报告 OR 撰写 OR 作者)) AND (M=(清单 OR 声明 OR 指导 OR 规范 OR 指南 OR 共识 OR 模板 OR 标准 OR 推荐意见))) OR (M=(CONSORT OR PRISMA OR STROBE OR SPIRIT OR STARD OR SRQR OR ARRIVE OR SQUIRE OR CHEERS OR TRIPOD OR COREQ))) AND (M=(体验 OR 访谈 OR 调查 OR 问卷调查 OR 焦点群众 OR 焦点小组 OR 质性研究 OR 定性研究))

### OSF

URL: <https://osf.io/>

Search Date: 15/12/2021

title:(((reporting OR writ* OR author*) AND (checklist* OR statement* OR guid* OR template* OR standard* OR recommendation* OR experience* OR interview* OR survey* OR questionnaire* OR "focus group*" OR facilitat* OR barrier* OR qualitative*)))

### Methods in Research on Research

URL: http://miror-ejd.eu/publications/

Search Date: 14/12/2021

JH manually searched the list of publications.

## Supplement 2: PRISMA-S Checklist

| \| No. \| Item \| Description \| Location \| \| --- \| --- \| --- \| --- \| \| 1 \| Database name \| Name each individual database searched, stating the platform for each. \| See Methods [Table 1](#tbl-info-sources) and Supplement 1 \| \| 2 \| Multi-database searching \| If databases were searched simultaneously on a single platform, state the name of the platform, listing all of the databases searched. \| See Methods [Table 1](#tbl-info-sources) and Supplement 1 \| \| 3 \| Study registries \| List any study registries searched. \| See Methods [Table 1](#tbl-info-sources) and Supplement 1 \| \| 4 \| Online resources and browsing \| Describe any online or print source purposefully searched or browsed (e.g., tables of contents, print conference proceedings, web sites), and how this was done. \| See Methods [Table 1](#tbl-info-sources) and Supplement 1 \| \| 5 \| Citation searching \| Indicate whether cited references or citing references were examined, and describe any methods used for locating cited/citing references (e.g., browsing reference lists, using a citation index, setting up email alerts for references citing included studies). \| See Methods, Approach to searching and data sources \| \| 6 \| Contacts \| Indicate whether additional studies or data were sought by contacting authors, experts, manufacturers, or others. \| See Methods, Approach to searching and data sources \| \| 7 \| Other methods \| Describe any additional information sources or search methods used. \| No other methods \| \| 8 \| Full search strategies \| Include the search strategies for each database and information source, copied and pasted exactly as run. \| Supplement 1 \| \| 9 \| Limits and restrictions \| Specify that no limits were used, or describe any limits or restrictions applied to a search (e.g., date or time period, language, study design) and provide justification for their use. \| See Methods, Inclusions and exclusion criteria. \| \| 10 \| Search filters \| Indicate whether published search filters were used (as originally designed or modified), and if so, cite the filter(s) used. \| See Methods, Electronic search strategy \| \| 11 \| Prior work \| Indicate when search strategies from other literature reviews were adapted or reused for a substantive part or all of the search, citing the previous review(s). \| See Methods, Electronic search strategy \| \| 12 \| Updates \| Report the methods used to update the search(es) (e.g., rerunning searches, email alerts). \| See Methods, Electronic search strategy \| \| 13 \| Dates of searches \| For each search strategy, provide the date when the last search occurred. \| See Methods, [Table 1](#tbl-info-sources) \| \| 14 \| Peer review \| Describe any search peer review process. \| See Methods, Electronic Search Strategy & Supplement 1 \| \| 15 \| Total records \| Document the total number of records identified from each database and other information sources. \| See Results, [Figure 1](#fig-prisma-flow-diagram) \| \| 16 \| Deduplication \| Describe the processes and any software used to deduplicate records from multiple database searches and other information sources. \| See Methods [Table 1](#tbl-info-sources) \| |
| --- | --- | --- | --- | --- | --- | --- | --- | --- | --- | --- | --- | --- | --- | --- | --- | --- | --- | --- | --- | --- | --- | --- | --- | --- | --- | --- | --- | --- | --- | --- | --- | --- | --- | --- | --- | --- | --- | --- | --- | --- | --- | --- | --- | --- | --- | --- | --- | --- | --- | --- | --- | --- | --- | --- | --- | --- | --- | --- | --- | --- | --- | --- | --- | --- | --- | --- | --- | --- |

##

## Supplement 3: ENTREQ Checklist

| \| No. \| Item \| Description \| Location \| \| --- \| --- \| --- \| --- \| \| 1 \| Aim \| State the research question the synthesis addresses. \| See Introduction, para. 4 \| \| 2 \| Synthesis methodology \| Identify the synthesis methodology or theoretical framework which underpins the synthesis, and describe the rationale for choice of methodology (e.g. meta-ethnography, thematic synthesis, critical interpretive synthesis, grounded theory synthesis, realist synthesis, meta-aggregation, meta-study, framework synthesis). \| See Methods, Synthesis Methodology para. 1 \| \| 3 \| Approach to searching \| Indicate whether the search was pre-planned (comprehensive search strategies to seek all available studies) or iterative (to seek all available concepts until they theoretical saturation is achieved). \| See Methods, Approach to Searching \| \| 4 \| Inclusion criteria \| Specify the inclusion/exclusion criteria (e.g. in terms of population, language, year limits, type of publication, study type). \| See Methods, Inclusion and Exclusion criteria \| \| 5 \| Data sources \| Describe the information sources used (e.g. electronic databases (MEDLINE, EMBASE, CINAHL, psycINFO, Econlit), grey literature databases (digital thesis, policy reports), relevant organisational websites, experts, information specialists, generic web searches (Google Scholar) hand searching, reference lists) and when the searches conducted; provide the rationale for using the data sources. \| See Methods [Table 1](#tbl-info-sources) \| \| 6 \| Electronic Search strategy \| Describe the literature search (e.g. provide electronic search strategies with population terms, clinical or health topic terms, experiential or social phenomena related terms, filters for qualitative research, and search limits). \| Supplement 1 \| \| 7 \| Study screening methods \| Describe the process of study screening and sifting (e.g. title, abstract and full text review, number of independent reviewers who screened studies). \| See Methods, Screening \| \| 8 \| Study characteristics \| Present the characteristics of the included studies (e.g. year of publication, country, population, number of participants, data collection, methodology, analysis, research questions). \| See Results, [Table 3](#tbl-study-characteristics) \| \| 9 \| Study selection results \| Identify the number of studies screened and provide reasons for study exclusion (e,g, for comprehensive searching, provide numbers of studies screened and reasons for exclusion indicated in a figure/flowchart; for iterative searching describe reasons for study exclusion and inclusion based on modifications t the research question and/or contribution to theory development). \| See Results, [Figure 1](#fig-prisma-flow-diagram) \| \| 10 \| Rationale for appraisal \| Describe the rationale and approach used to appraise the included studies or selected findings (e.g. assessment of conduct (validity and robustness), assessment of reporting (transparency), assessment of content and utility of the findings). \| See Methods, Describing and appraising records \| \| 11 \| Appraisal items \| State the tools, frameworks and criteria used to appraise the studies or selected findings (e.g. Existing tools: CASP, QARI, COREQ, Mays and Pope; reviewer developed tools; describe the domains assessed: research team, study design, data analysis and interpretations, reporting). \| See Methods, Describing and appraising records \| \| 12 \| Appraisal process \| Indicate whether the appraisal was conducted independently by more than one reviewer and if consensus was required. \| See Methods, Describing and appraising records \| \| 13 \| Appraisal results \| Present results of the quality assessment and indicate which articles, if any, were weighted/excluded based on the assessment and give the rationale. \| See Results, Search and [Table 3](#tbl-study-characteristics) \| \| 14 \| Data extraction \| Indicate which sections of the primary studies were analysed and how were the data extracted from the primary studies? (e.g. all text under the headings “results /conclusions” were extracted electronically and entered into a computer software). \| See Methods, Synthesis Methodology, para. 2 \| \| 15 \| Software \| State the computer software used, if any. \| See Methods, Synthesis Methodology, para. 2 \| \| 16 \| Number of reviewers \| Identify who was involved in coding and analysis. \| See Methods, Synthesis Methodology, para. 2 \| \| 17 \| Coding \| Describe the process for coding of data (e.g. line by line coding to search for concepts). \| See Methods, Synthesis Methodology, para. 2 \| \| 18 \| Study comparison \| Describe how were comparisons made within and across studies (e.g. subsequent studies were coded into pre-existing concepts, and new concepts were created when deemed necessary). \| See Methods, Synthesis Methodology, para. 2 \| \| 19 \| Derivation of themes \| Explain whether the process of deriving the themes or constructs was inductive or deductive. \| See Methods, Synthesis Methodology, para. 2 \| \| 20 \| Quotations \| Provide quotations from the primary studies to illustrate themes/constructs, and identify whether the quotations were participant quotations of the author’s interpretation. \| See Results, Synthesis Findings \| \| 21 \| Synthesis output \| Present rich, compelling and useful results that go beyond a summary of the primary studies (e.g. new interpretation, models of evidence, conceptual models, analytical framework, development of a new theory or construct). \| See Results, Synthesis Findings \| |
| --- | --- | --- | --- | --- | --- | --- | --- | --- | --- | --- | --- | --- | --- | --- | --- | --- | --- | --- | --- | --- | --- | --- | --- | --- | --- | --- | --- | --- | --- | --- | --- | --- | --- | --- | --- | --- | --- | --- | --- | --- | --- | --- | --- | --- | --- | --- | --- | --- | --- | --- | --- | --- | --- | --- | --- | --- | --- | --- | --- | --- | --- | --- | --- | --- | --- | --- | --- | --- | --- | --- | --- | --- | --- | --- | --- | --- | --- | --- | --- | --- | --- | --- | --- | --- | --- | --- | --- | --- |
